# High-fiber diet reduces bone formation but does not affect bone microarchitecture in type 2 diabetes individuals

**DOI:** 10.1093/jbmrpl/ziae111

**Published:** 2024-08-16

**Authors:** Malak Faraj, Giulia Leanza, Johannes Krug, Francesca Cannata, Viola Viola, Biagio Zampogna, Fabrizio Russo, Giuseppe Banfi, Giovanni Lombardi, Gianluca Vadalà, Laura Mangiavini, Rocco Papalia, Vincenzo Denaro, Björn Busse, Nicola Napoli

**Affiliations:** Research Unit of Endocrinology and Diabetes, Campus Bio-Medico University of Rome, 00128 Rome, Italy; Research Unit of Endocrinology and Diabetes, Campus Bio-Medico University of Rome, 00128 Rome, Italy; Department of Osteology and Biomechanics, University Medical Center Hamburg Eppendorf, 20246 Hamburg, Germany; Research Unit of Endocrinology and Diabetes, Campus Bio-Medico University of Rome, 00128 Rome, Italy; Research Unit of Endocrinology and Diabetes, Campus Bio-Medico University of Rome, 00128 Rome, Italy; Department of Orthopedic and Trauma Surgery, Departmental Faculty of Medicine and Surgery, Campus Bio-Medico University of Rome, 00128 Rome, Italy; Department of Orthopedic and Trauma Surgery, Departmental Faculty of Medicine and Surgery, Campus Bio-Medico University of Rome, 00128 Rome, Italy; Laboratory of Experimental Biochemistry and Molecular Biology, IRCCS Ospedale Galeazzi-Sant'Ambrogio, 20157 Milan, Italy; Vita-Salute San Raffaele University, 20132 Milan, Italy; Laboratory of Experimental Biochemistry and Molecular Biology, IRCCS Ospedale Galeazzi-Sant'Ambrogio, 20157 Milan, Italy; Department of Athletics, Strength and Conditioning, Poznań University of Physical Education, 61-871 Poznań, Poland; Department of Orthopedic and Trauma Surgery, Departmental Faculty of Medicine and Surgery, Campus Bio-Medico University of Rome, 00128 Rome, Italy; Laboratory of Experimental Biochemistry and Molecular Biology, IRCCS Ospedale Galeazzi-Sant'Ambrogio, 20157 Milan, Italy; Department of Biomedical Sciences for Health, University of Milan, 20133 Milan, Italy; Department of Orthopedic and Trauma Surgery, Departmental Faculty of Medicine and Surgery, Campus Bio-Medico University of Rome, 00128 Rome, Italy; Department of Orthopedic and Trauma Surgery, Departmental Faculty of Medicine and Surgery, Campus Bio-Medico University of Rome, 00128 Rome, Italy; Department of Osteology and Biomechanics, University Medical Center Hamburg Eppendorf, 20246 Hamburg, Germany; Interdisciplinary Competence Center for Interface Research, University Medical Center Hamburg Eppendorf, 20246 Hamburg, Germany; Research Unit of Endocrinology and Diabetes, Campus Bio-Medico University of Rome, 00128 Rome, Italy; Operative Research Unit of Osteometabolic and Thyroid Diseases, Fondazione Policlinico Universitario Campus Bio-Medico, 00128 Rome, Italy

**Keywords:** high-fiber diet, glucose control, type 2 diabetes, bone turnover, bone microarchitecture, Wnt signaling

## Abstract

Bone fragility is a recognized complication of type 2 diabetes mellitus (T2DM), increasing patient morbidity. Thus, the development of an effective intervention to prevent diabetic bone fragility is urgently needed. As lifestyle intervention represents an effective option for diabetes management, it may have an impact on bone health. While studies have shown a beneficial effect of dietary fiber in T2DM management, its effect on bone health is still unclear. Thus, we investigated the impact of a high-fiber diet on bone and glucose control in men and women with T2DM. Forty-five T2DM patients (HbA1c: 6.5% ± 0.49%, age: 74 ± 7.29 yr) scheduled for hip arthroplasty were randomly assigned to follow a high-fiber diet (38 g/day) or to make no diet changes for 12 wk. Interestingly, BMI decreased by 4% (*p* <.0001) and HbA1c by 3.4% (*p* <.0001) in the high-fiber diet group, but did not decrease in the control group. However, serum concentration of the bone formation marker procollagen type 1 amino-terminal propeptide (P1NP) decreased by 8.6 % in the high-fiber diet group (*p* =.0004), whereas it remained unchanged in the control group. In contrast, similar to the control group, serum concentration of the bone resorption marker C-terminal telopeptide of type I collagen (CTX) concentrations did not change in the high-fiber diet group. Bone microCT analysis revealed no changes in trabecular and cortical bone parameters between the high-fiber diet and control groups. Similarly, real-time (RT)-PCR analysis in bone tissue showed no changes in the gene expression of Wnt pathway-related genes (Sost, Dkk-1, Wnt10b, and Lef-1), bone formation markers (Runx2, Col1a1, and Ocn), and inflammatory cytokines (IL-6, IL-8, TNF-α, and IL-10) between the two groups. Our findings suggest that 12-wk high-fiber diet intervention improves metabolic outcomes in patients with T2DM. However, it may reduce bone formation without affecting bone microarchitecture or Wnt pathway regulation.

## Graphical Abstract

**Graphical Abstract:**
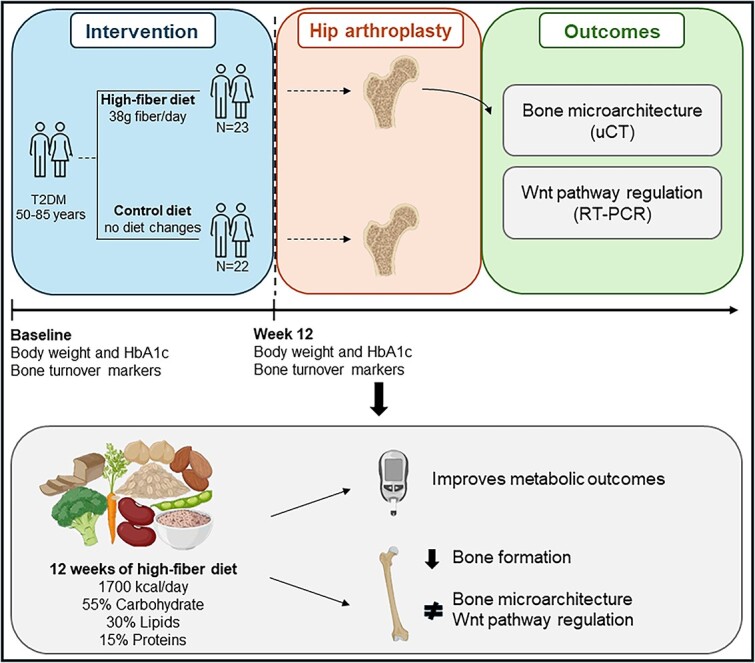

## Introduction

Type 2 diabetes mellitus (T2DM) is a chronic metabolic disease whose prevalence is increasing worldwide with the growth of the aging population, sedentary lifestyle, and obesity.^1^ T2DM has been identified as a novel risk factor for the skeleton. Fracture risk is increased in patients with T2DM despite having normal or high bone mineral density.^2^^,^^3^ Hence, it is clinically important to develop effective interventions to improve bone quality in T2DM.

Nutritional therapy aiming to improve glucose control is crucial for T2DM management and to reduce the risk of diabetes complications. Consuming dietary fiber is highly recommended for patients with T2DM.^4^^,^^5^ Dietary fiber is defined by the Institute of Medicine as a non-digestible carbohydrate and lignin that are found in plant-based foods.^6^ Studies have shown that higher fiber intake was associated with lower HbA1c, fasting plasma glucose, and insulin resistance in T2DM patients.^7–9^ Furthermore, dietary fiber is also rich in phenolic compounds that are known to reduce inflammation, a risk factor for diabetes.^10^^,^^11^ However, the effect of dietary fiber on bone loss in T2DM is unclear.

The canonical Wnt signaling pathway plays an important role in bone homeostasis as Wnt activation increases bone formation and reduces bone resorption.^12^ Sclerostin (SOST) and Dickkopf-1 (DKK-1) are two important inhibitors of Wnt signaling that impair osteoblast differentiation. Interestingly, in clinical studies, sclerostin levels were shown to be increased in T2DM,^13–15^ positively correlated with glycated hemoglobin (HbA1c), and inversely associated with bone formation markers. Moreover, we recently showed increased SOST and decreased runt-related transcription factor 2 (RUNX2) gene expression in bone tissue of T2DM patients.^16^ Besides elevated sclerostin, DKK-1 has also been shown to be increased in patients with T2DM.^17^^,^^18^ Therefore, the inhibition of Wnt signaling during hyperglycemia seems to be the link between hyperglycemia and reduced bone formation. Thus, interventions that improve glucose metabolism might concurrently improve osteogenesis in diabetes. In this study, we hypothesized that the beneficial effect of dietary fiber on glucose control might improve bone health in T2DM patients through a positive modulation of Wnt pathway. We demonstrated that 12 wk of a high-fiber diet improves metabolic outcomes but may reduce bone formation without altering bone microarchitecture or Wnt pathway regulation in patients with T2DM.

## Material and methods

### Study participants

Participants were recruited for this study between 2021 and 2023 at the Orthopedics departments of Galeazzi Institute and Policlinico Campus Bio-Medico of Rome. Patients affected by osteoarthritis who were scheduled for hip arthroplasty were screened for participation in this study. Men and women with T2DM, aged between 50 and 85 yr old, and with glycated hemoglobin ≥6.5% at the time of enrolment were included. Participants with any bone disease (such as osteoporosis, osteogenesis imperfecta, fibrous dysplasia, or malignancy) or calcium disorders, hepatic or renal disease were excluded. In addition, participants were excluded if they were on any medical treatment that affect bone metabolism (eg teriparatide, romosozumab, raloxifene, bisphosphonates, denosumab, thiazolidinediones, glucocorticoids, and anabolic steroids) or if they were current smokers. All procedures were conducted in accordance with the declaration of Helsinki and were approved by the Ethical Committee of the Campus Bio-Medico University of Rome. Written informed consent was obtained from all participants.

### Study design

**Figure 1:**
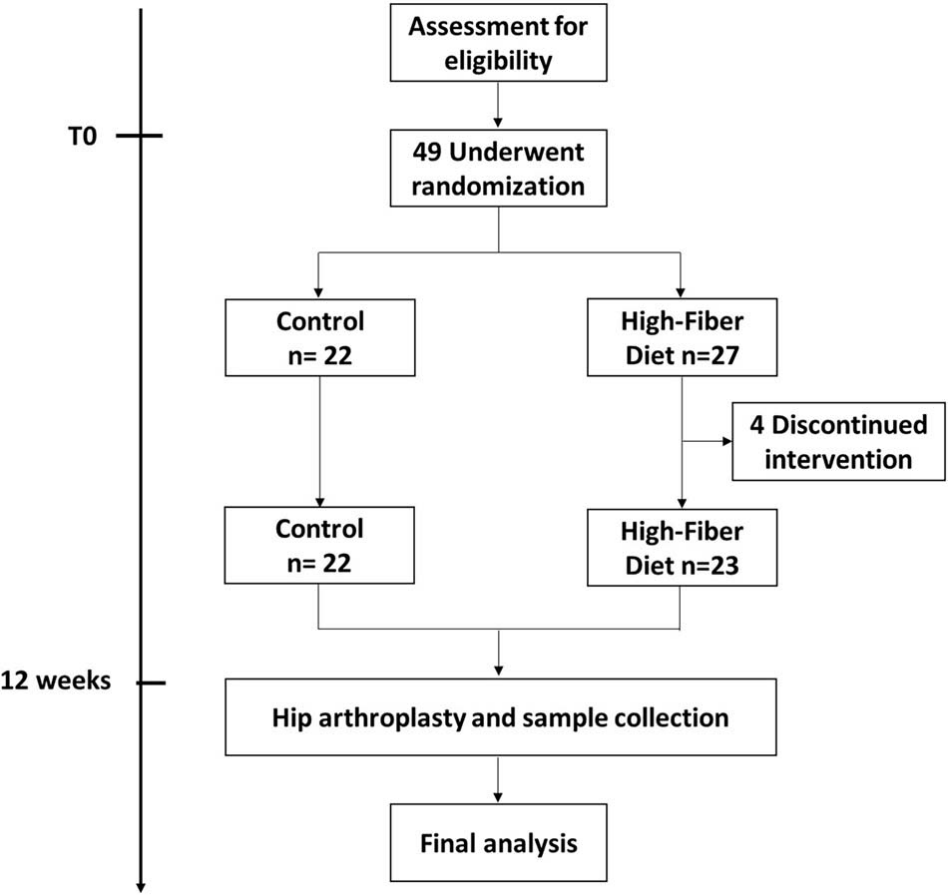
Screening, randomization, and follow-up.

At the baseline visit (12 wk before the hip arthroplasty), participants were randomized to follow a high-fiber diet (*n* = 23) or to make no diet changes (Control group, *n* = 22) for 12 wk. Participants in the control group were provided general information about a healthy diet during visits with the study dietitian, but they did not receive any advice to change their diet. However, participants in the diet group were prescribed a balanced diet with controlled micronutrients and a restricted caloric content of 1700 kcal/day. The macronutrient composition for the high-fiber diet was 55% of carbohydrates, 30% of lipids, and 15% of proteins, and contained 38 g of dietary fiber per day. Added sugars were not included. Compliance to the diet was investigated through a monthly follow-up visit with the study dietitian and a weekly telephone call. The study design is summarized in Figure 1.

### Diabetes status, anthropometric, and biochemical assessments

Diabetes status, current medications use, and disease history were collected from electronic medical records.

Body weight was measured at baseline and at 12 wk using a standard weighing scale. BMI was calculated as weight (kilograms [kg]) divided by the square of the height (meter [m]) (kg/m^2^).

Fasting morning blood samples were collected and analyzed by standard methods at baseline and after 12 wk for the measurement of HbA1c. Serum levels of procollagen type 1 amino-terminal propeptide (P1NP) and C-terminal telopeptide of type I collagen (CTX) were measured at baseline and after 12 wk using enzyme-linked immunosorbent assay (ELISA) kits according to the manufacturer’s protocols (Immunodiagnostic Systems, Frankfurt/Main, Germany).

### Bone samples

Femur specimens of the study participants were collected during the hip arthroplasty after 12 wk of dietary intervention. Trabecular bone from femoral head specimens was collected fresh and transferred to a tube containing sterile phosphate-buffered saline (PBS) 1×. Samples were washed multiple times in PBS to flush the bone marrow. Bone samples were snap-frozen and stored at −80 °C before RNA extraction. Moreover, bone cores (10–15 mm in diameter and 10–20 mm in length) were drilled from the inferomedial femoral neck, fixed in 4% paraformaldehyde for 3 days, and then stored in PBS at 4 °C until uCT analysis.

### Micro-computed tomography analysis of bone

The bone mass and microstructure of the inferomedial femoral neck were evaluated using micro-computed tomography (μCT 40, Scanco Medical AG, Switzerland). Hence, the bone cores were placed in a sample holder and fixed with sponges to prevent movement during scanning. The scanning settings were standardized to 55 kV, 145 μA, and 200 ms integration time, resulting in image stacks with 15-μm isotropic voxel size. For evaluation, the cortical and trabecular bone regions of interest were contoured manually, while the transitional zone and the damaged border regions were excluded from the analysis. Cortical bone was binarized using a global threshold of 550 mg HA/cm^3^ to assess cortical porosity (Ct.Po, %), cortical thickness (Ct.Th, mm), and cortical tissue mineral density (Ct.TMD, mg HA/cm^3^), and a global threshold of 450 mg HA/cm^3^ was applied to the trabecular bone to determine bone volume per total volume (BV/TV, %), trabecular thickness (Tb.Th, mm), trabecular separation (Tb.Sp, mm), and trabecular bone mineral density (Tb.BMD, mg HA/cm^3^) using XamFlow software (Lucid Concept AG, Zurich, Switzerland). Thickness and spacing measurements were performed through an implementation of the model independent volume-based assessment method proposed by *Hildebrand and Rüegsegger.*^19^

### RNA extraction, cDNA synthesis, and quantitative real-time PCR

Total RNA was extracted from trabecular bone tissue using TRIzol reagent (Thermo Fisher Scientific) according to the manufacturer’s instructions.^20^ Quantification of the RNA was assessed spectrophotometrically (TECAN, InfiniteM200PRO), and only samples with a 260/280 absorbance ratio (A260/A280) between 1.8 and 2 were used for reverse transcription, and 1 μg of RNA was reverse transcribed using a High-Capacity cDNA Reverse Transcription Kit (Applied Biosystems) according to product protocol (25 °C for 10 min, 37 °C for 2 h, 85 °C for 5 min) followed by Taqman-based quantitative RT-PCR at standard cycling conditions (95 °C for 10 min; 40 cycles of 95 °C for 15 s and 60 °C for 1 min; followed by 95 °C for 15 s, 60 °C for 15 s, and 95 °C for 15 s). Used Taqman probes (ThermoFisher Scientific) are listed in Table SS1. The results were calculated based on the ΔCT method and were presented as relative expression normalized to the *β-Actin* level.

### Statistical analysis

Statistical analyses were performed using the statistical software GraphPad Prism 9 (GraphPad Software, CA, USA). Normal distribution was tested using the Shapiro–Wilk normality test. Patients’ characteristics were represented as number (percentage) for categorical variables and mean ± SD for continuous variables. Group data are presented in boxplots with median and interquartile range; whiskers represent maximum and minimum values. Student’s *t*-test was used to compare normally distributed data, and a Mann–Whitney nonparametric test otherwise. Two-way ANOVA followed by Bonferroni’s multiple comparison test was used to compare two groups across multiple time points. Spearman’s correlation was used to examine the relationships between changes in bone turnover markers and changes in body weight. Two-tailed *p*-value <.05 was considered statistically significant.

## Results

### Study population

Participants’ characteristics at baseline are shown in Table 1. A total of 45 T2DM subjects were randomized to a diet intervention for 12 wk: standard diet recommendation (control, *n* = 22) and high-fiber diet (*n* = 23). The mean age of the participants was 74 ± 7.29 yr and the mean HbA1c was 6.5% ± 0.49%. Most participants were females (67%). There were no significant differences between the two groups in baseline characteristics including age, BMI, and HbA1c.

**Table 1:** Baseline characteristics of study participants.

**T2DM subjects (*n* = 45)**			
	**Control diet (*n* = 22)**	**High-fiber diet (*n* = 23)**	***p*-value**
**Age (yr)**	74.9 ± 7.07	73.9 ± 7.62	0.77
**Females, *n* (%)**	18 (82%)	12 (52%)	0.03
**BMI (kg/m^2^)**	28.82 ± 2.14	29.85 ± 3.03	0.13
**HbA1c (%)**	6.49 ± 0.45	6.50 ± 0.53	0.98

Data are presented as mean ± SD or as otherwise indicated. Abbreviation: HbA1c = glycated haemoglobin.

### Effect of a high-fiber diet on metabolic parameters

**Figure 2:**
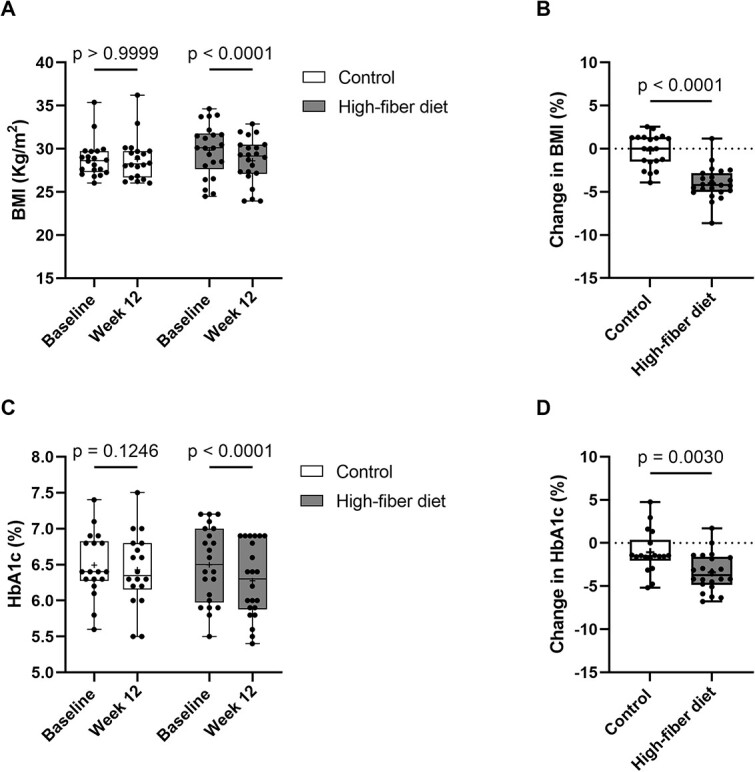
High-fiber diet improves metabolic parameters. (A) BMI at baseline and after 12 wk of follow-up in the control and high-fiber diet group. (B) Percentage changes from baseline in BMI in the control and high-fiber diet group during the 12-wk diet intervention. (C) Glycated hemoglobin (HbA1c) at baseline and after 12 wk of follow-up in the control and high-fiber diet group. (D) Percentage changes from baseline in HbA1c in the control and high-fiber diet group during the 12-wk intervention. Data are presented as median and interquartile range (control: *n* = 22, high-fiber diet: *n* = 23). Statistical difference was determined by two-way ANOVA (A and C) and unpaired Student’s *t*-test (B and D).

BMI was significantly reduced at 12 wk in the high-fiber diet group compared with baseline (29.85 ± 3.03 Kg/m^2^ vs 28.08 ± 3.62 Kg/m^2^, *p* <.0001), but not in the control group (28.82 ± 2.14 Kg/m^2^ vs 28.78 ± 2.47 Kg/m^2^, *p* >.999) (Figure 2A). Group comparison revealed a significant difference in the changes in BMI in the high-fiber diet group (−4% ± 1.89%) compared with the control group (−0.20% ± 1.90%) (*p* <.0001) during the 12-wk follow-up (Figure 2B).

Interestingly, glycated hemoglobin (HbA1c), which reflects the average blood glucose levels over the past 2–3 mo, was significantly reduced at 12 wk in the high-fiber diet group compared with baseline (6.50% ± 0.53% vs 6.28% ± 0.51%, *p* <.0001), but not in the control group (6.49% ± 0.45% vs 6.43% ± 0.51%, *p* =.124) (Figure 2C). Group comparisons revealed a significant difference in the changes in HbA1c levels in the high-fiber diet group (−3.40% ± 2.16%) compared with the control group (−1.07% ± 2.48%) (*p* =.003) during the 12-wk follow-up (Figure 2D).

### Effect of a high-fiber diet on bone turnover markers in T2DM

**Figure 3:**
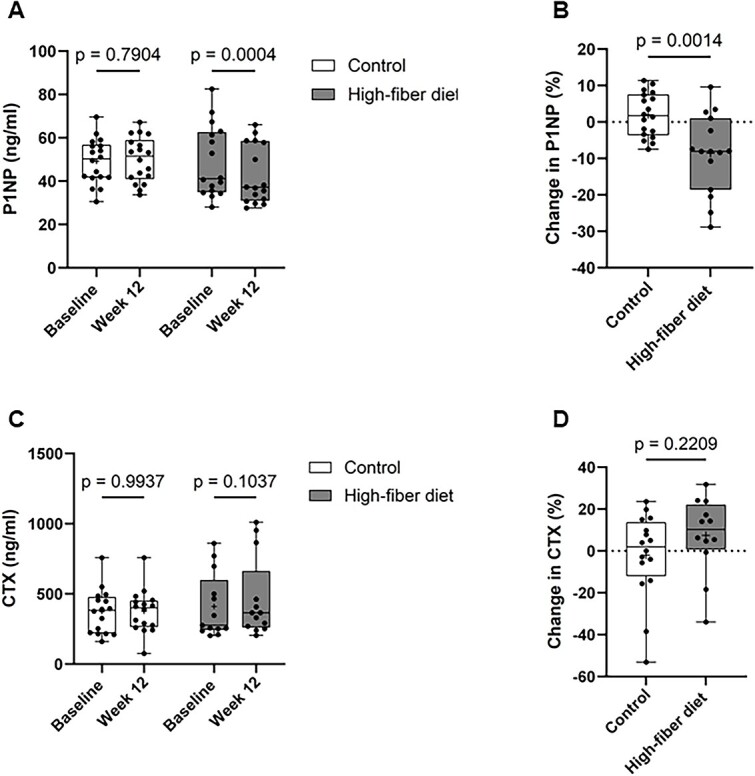
High-fiber diet reduces bone formation in T2DM patients. Seum procollagen type 1 amino-terminal propeptide (P1NP) at baseline and after 12 wk of follow-up in the control and high-fiber diet group (control: *n* = 18, high-fiber diet: *n* = 16). (B) Percentage changes from baseline in P1NP in the control and high-fiber diet group during the 12-wk diet intervention. (C) Serum C-terminal telopeptide of type I collagen (CTX) at baseline and after 12 wk of follow-up in the control and high-fiber diet group (control:16, high-fiber diet: 13). (D) Percentage changes from baseline in CTX in the control and high-fiber diet group during the 12-wk diet intervention. Data are presented as median and interquartile range. Statistical difference was determined by two-way ANOVA (A and C) and unpaired Student’s *t*-test (B and D).

We next addressed whether 12 wk of a high-fiber diet could impact bone metabolism. Serum concentration of the bone formation marker P1NP significantly decreased at 12 wk in the high-fiber diet group compared with baseline (48.89 ± 16.46 ng/ml vs 42.79 ± 13.61 ng/ml, *p* =.0004), but not in the control group (49.12 ± 10.34 ng/ml vs 49.97 ± 10.35 ng/ml, *p* =.79) (Figure 3A). In turn, changes in serum P1NP were significantly lower in high-fiber diet group (−8.59% ± 10.88%) compared with the control group (1.96% ± 6.03%) (*p* =.001) during the 12-wk follow-up (Figure 3B). In contrast, the high-fiber diet tended to increase the serum concentration of the bone resorption marker CTX, but this change was not statistically significant (Figure 3C, D).

**Figure 4:**
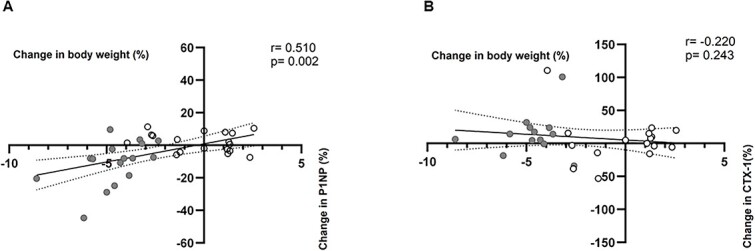
Changes in procollagen type 1 amino-terminal propeptide (P1NP) correlate with changes in body weight. (A) Spearman correlation of the changes in P1NP concentration and body weight during the 12-wk diet intervention. (B) Spearman correlation of the changes in C-terminal telopeptide of type I collagen (CTX) concentration and body weight during the 12-wk diet intervention.

Spearman correlation analysis revealed a positive correlation between changes in body weight and changes in serum P1NP (*r* = 0.51, *p* =.002) (Figure 4A). In comparison, there was no significant correlation between changes in body weight and changes in serum CTX (Figure 4B).

### Effect of a high-fiber diet on bone microarchitecture in T2DM

**Figure 5:**
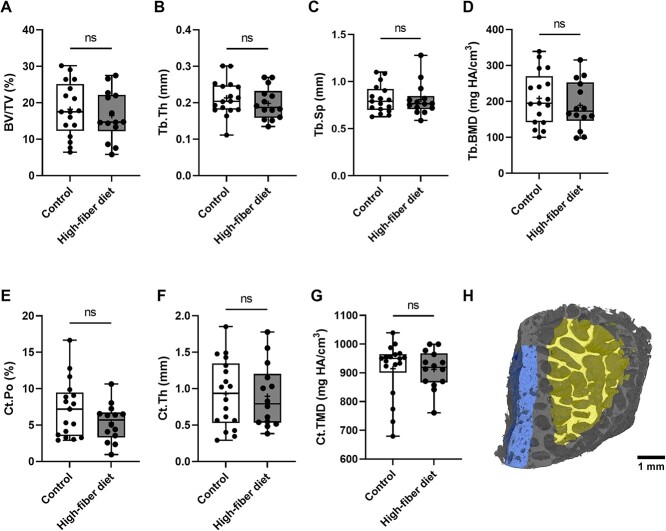
High-fiber diet does not affect bone microarchitecture in T2DM patients. Femoral inferomedial neck bone of T2DM patients after 12 wk on a high-fiber diet was analyzed by microCT. Trabecular bone parameters were evaluated as (A) bone volume per total volume (BV/TV), (B) trabecular thickness (Tb.Th), (C) trabecular separation (Tb.Sp), and (D) trabecular bone mineral density (Tb.BMD). Cortical bone parameters were evaluated as (E) cortical porosity (Ct.Po), (F) cortical thickness (Ct.Th), and (G) cortical tissue mineral density (Ct.TMD). (H) Representative reconstruction of trabecular and cortical bone. Blue represents cortical region and yellow represents trabecular region. Data are presented as median and interquartile range (control: *n* = 18, high-fiber diet: *n* = 14). The *p* values were determined by Mann–Whitney test.

Because of the lack of trabecular region in one biopsy, 31 biopsies (Control: *n* = 17, high-fiber diet: *n* = 14) were used for trabecular and 32 biopsies (Control: *n* = 18, high-fiber diet: *n* = 14) for cortical analysis. After 12 wk of a high-fiber diet, micro-CT scans of inferomedial femoral neck specimens showed no significant changes in trabecular (BV/TV, Tb.Th, Tb.Sp, and Tb.BMD) and cortical parameters (Ct.Po, Ct.Th, and Ct.TMD) between the two groups (Figure 5).

### Effect of a high-fiber diet on Wnt signaling regulation and bone formation in T2DM

**Figure 6:**
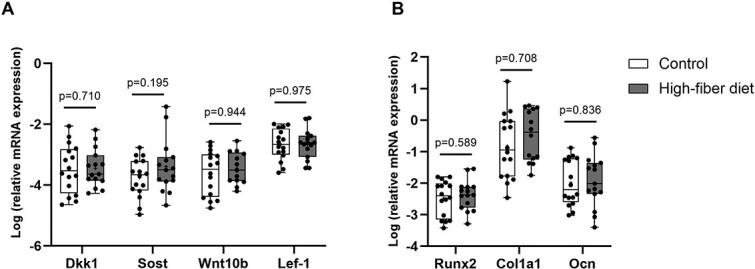
Wnt pathway regulation is not affected by high-fiber diet in T2DM patients. (A) Gene expression of dickkopf-1 (*Dkk1*), sclerostin (*SOST*), Wnt family member 10B (*Wnt10b*), lymphoid enhancer-binding factor 1 (*Lef1*), (B) runt-related transcription factor 2 (*Runx2*), collagen type I alpha 1 chain (*Col1a1*), and osteocalcin (*Ocn*) in bone of T2DM patients following 12-wk of high-fiber diet. Gene expression levels were normalized to β-actin. Data are presented as logarithmic scale median and interquartile range (control: *n* = 16, high-fiber diet: *n* = 15). The *p* values were determined by unpaired Student’s *t*-test.

To investigate the molecular mechanisms underlying reduced bone formation in the high-fiber diet group, we examined the expression of Wnt pathway-related genes. No differences were observed in the gene expression of Sost, Dkk-1, Wnt10b, and Lef-1 between the high-fiber diet and control group (Figure 6A). Moreover, expression of the bone formation markers, Runx2, Col1a1, and Ocn was not affected by a high-fiber diet (Figure 6B).

### Effect of a high-fiber diet on inflammation in T2DM

**Figure 7:**
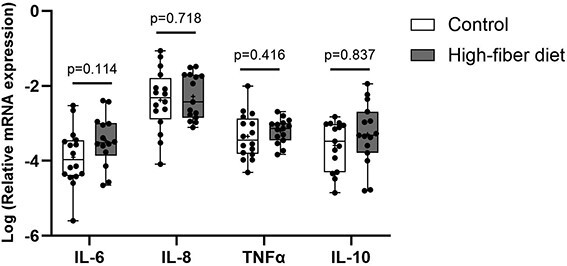
High-fiber diet does not affect inflammation in T2DM patients. Gene expression of inflammation markers (*IL-6*, *IL-8*, *TNFα*, and *IL-10*) in bone of T2DM patients following 12 wk of high-fiber diet. Gene expression levels were normalized to β-actin. Data are presented as logarithmic scale median and interquartile range (control: *n* = 16, high-fiber diet: *n* = 15). The *p* values were determined by unpaired Student’s *t*-test.

As dietary fiber is known to reduce inflammation, a risk factor for diabetic bone disease, we examined the expression of the inflammation markers IL-6, IL-8, TNF-α, and IL-10 in bone tissue. The expression of IL-6, IL-8, TNF-α, and IL-10 was not altered after a high-fiber diet (Figure 7).

## Discussion

In this study, we explored the effect of a high-fiber diet on diabetic bone disease among men and women with T2DM. Our results showed that a short-term (12 wk) high-fiber diet intervention led to beneficial effects on metabolic parameters. However, the high-fiber diet also reduced bone formation, as indicated by lower serum concentrations of P1NP. In contrast, bone microarchitecture and Wnt-pathway were not affected by high-fiber diet.

To the best of our knowledge, this is the first study to investigate the effect of a high-fiber diet on diabetic bone disease in humans. As expected, 12 wk of a high-fiber diet reduced BMI and HbA1c levels in T2DM patients, which is clinically relevant for the management of T2DM. Our findings are consistent with previous studies that showed improved glucose metabolism and reduced BMI with higher fiber intake in T2DM patients.^7–9^ These results are supported by the American Diabetes Association/European Association for the Study of Diabetes (ADA/EASD), which indicates that lifestyle modifications are crucial for T2DM management.^21^ The beneficial effects excreted by dietary fiber on glucose control, especially soluble ones, are most likely due to its ability to form viscous gels that delay gastric emptying and slow down macronutrients absorption in the small intestine, thus causing a feeling of satiety and reduction in postprandial glucose response.^22^ Additionally, higher fiber intake may improve glucose control by the production of short-chain fatty acids (SCFAs) during colonic fermentation. SCFA activates G protein-coupled receptors, particularly GPR41 and GPR43 on the brush border membrane, stimulating the secretion of satiety hormones GLP-1 and peptide YY from enteroendocrine L-cells and thereby controlling insulin secretion and glucose homeostasis.^23^^,^^24^

Contrary to our hypothesis, we found that despite the improvement in glucose control, a high-fiber diet decreased the serum concentration of the bone formation marker P1NP, while the bone resorption marker CTX was unaffected. Previous studies on the effect of high-fiber diet on bone turnover markers in nondiabetic human cohorts have shown inconsistent results, with some reporting no change,^25^ increases,^26^ or decreases^27^ in bone formation and resorption markers. These discrepancies could be explained by variations in subject characteristics, type and amounts of fiber, and intervention period, highlighting the importance of detailed investigations and standardized procedures. Our results indicate that a high-fiber diet contributes to reduced bone formation, which could have adverse effects on bone health in patients with T2DM. The reasons for this detrimental effect on bone are not fully elucidated. Notably, previous findings have shown that weight loss from diet alone is associated with bone loss in older adults.^28^^,^^29^ Consistent with this, we observed a positive correlation between changes in body weight and changes in P1NP concentrations, suggesting that weight loss may contribute to the decreased bone formation induced by the high-fiber diet. A common explanation for the bone loss induced by weight loss is the reduction in mechanical stress on the weight-bearing skeleton, which could influence bone turnover.^30^ In addition, our findings support the notion that high fiber intake may impair mineral absorption, especially calcium, which is controversially discussed in the literature. Although some studies have shown a negative effect of high fiber intake on calcium absorption in humans and rats,^31^^,^^32^ others have found no effect^33^ or even a positive effect.^34^^,^^35^ The ability of dietary fiber to chelate ions and from unabsorbable fiber-mineral complexes might impair calcium absorption. However, our study did not assess calcium levels, so reduced calcium absorption following a high-fiber diet cannot be excluded as one of the reasons for decreased bone formation.

Although 12 wk of a high-fiber diet reduced bone formation, it did not lead to alterations in bone microarchitecture or regulation of the Wnt pathway. Previous studies have reported inconsistent results regarding the impact of a high-fiber diet on bone in nondiabetic human cohorts. Although some studies reported increased BMD^36–38^ by high-fiber diet, others found no alterations.^27^^,^^39^ Such conflicting results may be related to differences in subject charetcteritcs, type and amount of fiber, and intervention duration. Of interest, data from the Framingham Offspring Cohort Study showed that dietary fiber was associated with lower femoral neck BMD in older men during an 8-yr follow-up.^36^ However, in a 2-yr randomized clinical trial, Slevin et al. reported that supplementation with prebiotic fiber did not affect femur or lumbar spine BMD in postmenopausal women.^27^ The absence of an effect of high-fiber diet on bone microarchitecture in our study could be due to the short duration of the intervention (12 wk). Thus, a longer intervention period would be required to further understand the effect of dietary fiber on bone quality in T2DM.

Our study has several limitations. A potential limitation is the short duration of the intervention due to the intensity of the intervention. More changes may have been seen with a longer duration of intervention. Another limitation is the small sample size. Finally, even though the participants were having face-to-face meetings with the study dietitian to assess their adherence to the diet, a potential for a measurement error may have occurred. However, this issue is common in most diet studies.

In conclusion, our findings suggest that short-term (12 wk) intake of a high-fiber diet is effective in improving metabolic outcomes in patients with T2DM. However, it may also reduce bone formation, with no observed effect on bone microarchitecture. Further long-term follow-up studies in larger patient cohorts are needed to understand the effect of a high-fiber diet on diabetic bone disease.

## Supplementary Material

Faraj_et_al_Supplemetary_Materials_ziae111

## Data Availability

The data generated or analyzed for this study are not publicly available due to patients’ privacy. The data will be shared on reasonable request to the corresponding author.
