# Supplementary material for: High-fiber diet reduces bone formation but does not affect bone microarchitecture in type 2 diabetes individuals
Source: JBMR Plus. 2024 Aug 16;8(10):ziae111. doi: 10.1093/jbmrpl/ziae111 (PMC11382141; doi:10.1093/jbmrpl/ziae111)
Supplement: Faraj_et_al_Supplemetary_Materials_ziae111 [file faraj_et_al_supplemetary_materials_ziae111.docx]

**Supplementary Table 1.** List of primers used for qRT-PCR.

| **Gene** | **Primer** |
| --- | --- |
| βAct | Hs01060665_g1 |
| Col1a1 | Hs00164004_m1 |
| Dkk1 | Hs00183740_m1 |
| IL-6 | Hs00174131_m1 |
| IL-8 | Hs00174103_m1 |
| IL-10 | Hs00961622_m1 |
| Lef-1 | Hs01547250_m1 |
| Ocn | Hs01587814_g1 |
| Runx2 | Hs01047973_m1 |
| Sost | Hs00228830_m1 |
| Tnfα | Hs00174128_m1 |
| Wnt10b | Hs00928823_m1 |

Gene abbreviations: βAct = beta actin, Col1a1 = Collagen type I alpha 1 chain, Dkk1 = Dickkopf-1, IL-6 = Interleukin 6, IL-8 = Interleukin 8, IL-10 = Interleukin 10, Lef-1 = Lymphoid enhancer-binding factor 1, Ocn = Osteocalcin, Runx2 = Runt-related transcription factor 2, Sost = Sclerostin, Tnfα = Tumor necrosis factor, Wnt10b= Wnt family member 10B.
